# Investigating the Ability to Mask Dental Discoloration by CAD/CAM Bleach Shade Ceramics in Different Thicknesses

**DOI:** 10.1002/cre2.70102

**Published:** 2025-03-02

**Authors:** Shervin Reybod, Fariba Ezoji, Ghazaleh Ahmadizenouz, Behnaz Esmaeili

**Affiliations:** ^1^ Student Research Committee Babol University of Medical Sciences Babol I.R. Iran; ^2^ Dental Materials Research center, Health Research Institute Babol University of Medical Sciences Babol I.R. Iran

**Keywords:** ceramic, computer‐aided designing/computer‐aided manufacturing (CAD/CAM), dental discoloration, thickness

## Abstract

**Objectives:**

The initial color of a ceramic restoration is determined by the background color. Dental ceramics are great at masking a variety of stains, which helps achieve a natural and beautiful smile. A recent study delved into the effectiveness of CAD/CAM bleach shade ceramics of varying thicknesses in concealing dental discoloration.

**Material and Methods:**

In this laboratory research, ceramic samples including feldspathic ceramics, lithium silicate ceramics, and zirconia ceramics of varying thicknesses were used. Each type of ceramic had a thickness of 0.5, 1.0, 1.5, and 2.0 mm, with dimensions of 7 × 7 mm (*n* = 10). Backgrounds of C4 and A2 color porcelain were used. To evaluate color coverage, ceramic pieces were placed on the C4 and A2 porcelain backgrounds, and the Vita spectrophotometer was used to calculate the L*a*b* parameters and color difference (ΔE00). A ΔE00 value of ≤ 1.8 was considered clinically acceptable. The data was analyzed using two‐way and one‐way analysis of variance tests, and pairwise comparisons of groups were performed using Tukey's test.

**Results:**

The research revealed that both the thickness and type of ceramic material significantly influenced the color changes of the samples, with their interaction also playing a crucial role (all three: *p* < 0.001). Zirconia demonstrated superior color masking performance on the C4 substrate at 0.5 and 1.0 mm thicknesses. The masking ability of ceramics varied at 0.5, 1.0, and 1.5 mm but notably improved at a thickness of 2.0 mm.

**Conclusions:**

Zirconia generally demonstrated superior masking ability across all thicknesses, while other ceramics exhibited commendable performance only at 1.5 and 2.0 mm thicknesses. Nevertheless, augmenting the thickness of ceramic restorations amplified their masking capability.

## 1 Introduction

The request for teeth whitening is frequently encountered in dental practices. Treatment options for discolored teeth include bleaching treatments for both vital and non‐vital teeth, microabrasion, porcelain and composite veneers, ceramic veneers, or a combination of these modalities (Shadman et al. 2015). Porcelain restorations are particularly favored due to their exceptional optical properties, optimal translucency, and superior esthetics, making them the preferred choice for patients and dental practitioners. This preference is attributed to the conservative nature of the technique and the minimal need for dental structure removal compared to alternative full‐coverage restorations (Xing et al. 2017).

The appearance and final result are carefully assessed based on several factors when evaluating ceramic restorations. These factors include the color of the underlying dental structure, the thickness of the ceramic layers, and the color of the cement used. One of the primary determinants of the appearance of a ceramic restoration is the underlying tooth structure. For example, if a ceramic restoration is placed on a dark tooth structure (such as an endodontically treated tooth), the color underneath the crown can have a significant impact. Particularly in the cervical areas, potentially affecting its overall esthetic. It is crucial to consider various elements such as ceramic thickness, ceramic color, and cement color to address these potential issues. The clinician can control the thickness of the ceramic to manage the final translucency of the restoration, whereas the color of the cement has a comparatively lesser effect (Chaiyabutr et al. 2011).

The thickness of the ceramic used for dental restorations is directly influenced by the extent of tooth grinding. For optimal results, it is ideal to have a thin and translucent ceramic material bonded to the tooth without any discoloration, creating a visually pleasing veneer restoration (Xing et al. 2017). To minimize the impact of the natural tooth structure on the color of the restoration, it is recommended that the ceramic utilized has a minimum thickness of 2.0 mm (Chaiyabutr et al. 2011).

The color masking capability of an all‐ceramic restoration plays a crucial role in selecting the appropriate type of ceramic material. This capability is typically assessed by measuring the color change (Δ*E*) after the restoration is exposed to different backgrounds. If a restoration system has perfect masking ability, there will be no discernible color difference (Δ*E* = 0). However, even if the color difference of a restoration is not perfect, it can still be considered visually acceptable based on esthetic standards if the required criteria (Δ*E* < 3.3) are met (Chu et al. 2007).

Ceramic materials are manufactured using various techniques, including sintering, condensation, casting, pressing, slip casting, and computer‐aided design and manufacturing (CAD/CAM). The CAD/CAM system has attracted increased attention due to several factors, including using new materials, reduced time and cost in the laboratory, and enhanced quality control capabilities (Kang et al. 2018). Various CAD/CAM materials are available, such as feldspathic ceramics, lithium disilicate, lithium silicate reinforced with zirconia, ceramics with resin penetration, and nanoceramic resin. Each of these materials possesses distinct chemical structures from one another (Sari et al. 2018).

The Vita Mark II (VITA Zahnfabrik, Germany) is a type of feldspathic ceramic. It is composed of 56%–64% silica (SiO_2_), 20%–23% alumina (Al_2_O_3_), 6%–9% sodium oxide (Na_2_O), and 6%–8% potassium oxide (K_2_O). This ceramic primarily contains feldspathic crystalline particles in a glassy matrix. It comes in various colors and is commonly used to create veneers, inlays, and onlays, as well as anterior and posterior single‐unit crowns (Straumann n.d.; Jafari et al. 2018).

Vita Suprinity PC ceramic (VITA Zahnfabrik, Germany) is a lithium silicate ceramic reinforced with zirconia. This ceramic material contains 56%–65% silica (SiO_2_), 8%–12% zirconia (ZrO_2_), and 11%–19% lithium silicate (Li_2_Si_2_O_5_). The presence of zirconia in this ceramic improves its mechanical properties. On the other hand, Celtra Duo (Dentsply Sirona Restorative, Germany) is a CAD/CAM ceramic block composed of zirconia‐enriched lithium silicate. This material contains 56%–64% silica (SiO_2_), 10%–18% lithium silicate (Li_2_Si_2_O_5_), and 8%–12% zirconia (ZrO_2_). Combining these components in Celtra Duo enhances the ceramics' mechanical properties and strength.

Zirconia (5Y‐TZP, Luxen SE, White, HT) is a type of ceramic block that is composed primarily of 90%–95% zirconia (ZrO_2_) and 5%–10% yttria (Y_2_O_3_). Yttria (Y_2_O_3_) functions as a stabilizing agent in this ceramic material, playing a crucial role in maintaining the zirconia in the tetragonal phase and preventing potential cracking (Dental Tribune 2015). It is important to note that these factors can significantly impact their effectiveness in providing color masking due to variations in the chemical composition, size, and crystal distribution in different ceramics.

In a study conducted by Zhang and Zhao in 2003, it was noted that Vita Mark II ceramic with thicknesses of 0.5, 1.0, and 1.5 mm demonstrated an excellent ability to conceal the color of its underlying structure (Zhang and Zhao 2003). Furthermore, Carneiro et al. (2018) investigated the effects of thickness on the translucency of four different types of ceramics: Vita Mark II, Suprinity, IPS e.max Press, and translucent zirconia. This study revealed that an increase in thickness led to a decrease in translucency for all ceramic samples. Specifically, Suprinity ceramic exhibited the highest translucency, while zirconia demonstrated the lowest translucency (Carneiro et al. 2018). Passos et al. (2019) studied the thickness variations of 1.0, 1.5, and 2.0 mm of lithium silicate ceramic reinforced with zirconia in a separate research study. Their findings indicated that a minimum thickness of 1.5 mm was required to mask the C2 substrate, and a thickness of 2.0 mm was necessary for the gold substrate (Passos et al. 2019).

At present, various ceramic systems are available in the market. However, there has been insufficient research regarding the impact of a discolored core on the final color of bleach shade ceramics. This study aimed to assess the masking capability of bleach shade ceramics at different thicknesses—0.5, 1.0, 1.5, and 2.0 mm—under laboratory conditions. The study's null hypothesis stated that different thicknesses of CAD/CAM bleach shade ceramics have the same masking abilities.

## 2 Materials and Methods

In this study, 40 samples of each of the following ceramic materials were prepared: Celtra Duo (CD), Vita Suprinity (VS), Vita Mark II (VM), and 5Y‐TZP Zirconia (Zr) (Shadman et al. 2015). In total, 160 bleach shade ceramic samples were used; their characteristics are presented in Table 1.

**Table 1:** Characteristics of bleach color ceramics.

Ceramic type	Color	Type	Manufacturing company
CD	BL2	Zirconia‐reinforced lithium silicate ZLS	Dentsply Sirona, Germany
VS	OM1	Zirconia‐reinforced lithium silicate ZLS	VITA Zahnfabrik, Germany
VM	OM1	Feldspathic Ceramic	VITA Zahnfabrik, Germany
Zr	White, HT	5Y‐ TZP Zirconia	Dental Max, Korea

### 2.1 Preparation of Ceramic Samples

The ceramic blocks (CD, VS, VM) were cut into square samples measuring 7 × 7 mm, with thicknesses of 0.5, 1, 1.5, and 2 mm, using a slow‐speed saw (Delta Precision Sectioning Machine, Mashhad, Iran). Plenty of water was applied during the cutting process.

5Y‐ TZP Zirconia discs were cut from blank zirconia and sintered in a Kousha Fan Pars dental furnace (auto‐sinter 1650 KFP, Iran) for 2 h at 1500°C. To account for the shrinkage of zirconia during sintering, the shrinkage coefficient (20% to 25%) was factored into the design process in the software. In total, 160 ceramic samples (*n* = 10) were prepared based on the thickness and ceramic type parameters (4 × 4 × 10 mm). The ceramic samples were then polished with 600‐grit silicon carbide paper. Using a Shinwa Digital Caliper (Niigata, Japan), the thickness of the ceramic samples was measured and confirmed to be 0.5 ± 0.05 mm, 1.0 ± 0.05 mm, 1.5 ± 0.05 mm, and 2.0 ± 0.05 mm, respectively.

### 2.2 Glazing

VITA Suprinity ceramics, according to the manufacturer's recommendation, require a crystallization step in the VITA furnace. During the crystallization step, the samples were glazed with VITA AKZENT Plus (VITA Zahnfabrik, Bad Sackingen, Germany) at a temperature of 820°C for 12 min. Vita Mark ceramics was glazed in the furnace at 950°C for 10 min with the VITA AKZENT Plus glaze application. In addition, Celtra Duo ceramic was glazed with CELTRA universal Glaze (Dentsply Sirona Restorative, Germany) at 820°C for 8 min. Zirconia ceramic was also glazed with Ceramill Glaze (AmannGirrbach, Austria) for 10 min at a temperature of 850°C. Finally, the square‐shaped samples were cleaned of fat and contamination using ultrasonication (BioSonic UC50D, Coltene, Whaledent, USA) for 5 min in a 99% ethanol solution.

### 2.3 Background Preparation

The substrate was prepared using porcelain in C4 and A2 colors (Shofu, Japan) with dimensions of 10 mm × 10 mm and a thickness of 3 mm.

### 2.4 Color Measurement

Color measurements were taken using a Vita Easyshade Advance 4.0 spectrophotometer (VITA Zahnfabrik, Bad Sackingen, Germany). This device records L*a*b* parameters. In this context, *L* refers to brightness, *a* represents the red‐green vector, and *b* represents the yellow‐blue vector.

The A2 ceramic color was designated as the standard color within each group. A silicone barrier was constructed around the Easy Shade head unit and the ceramic sample to mitigate the influence of ambient light and ensure the reproducibility of the colorimetric data.

To accurately determine the masking ability of the bleach shade ceramic samples, the following steps were taken: First, the samples were placed on the A2 background with a drop of water to prevent light from refracting between the ceramic and the background. The measuring device was then positioned at the center of each ceramic sample, and the *L*1, *a*1, and *b*1 values were recorded. Next, the bleach shade ceramic samples were placed on the C4 background, and the same procedure was repeated to measure the color parameters and record the *L*2, *a*2, and *b*2 values.

Each sample's color measurement was repeated three times. Before measuring each sample's color, the device was calibrated according to the manufacturer's instructions. Since only one operator performed the measurement, the use of kappa values was not necessary for this study. Finally, ΔE00 values were obtained using the CIEDE2000 formula (Sharma et al. 2005). The calculations were performed using the Colormine online tool to ensure precision and consistency in the obtained values.

CIEDE2000 formula:

$$∆E00=\sqrt{{\left(\frac{∆L^{\prime }}{K_{L}S_{L}}\right)}^{2}+{\left(\frac{∆C^{\prime }}{K_{C}S_{C}}\right)}^{2}+{\left(\frac{∆H^{\prime }}{K_{H}S_{H}}\right)}^{2}+R_{T}\left(\frac{∆C^{\prime }}{K_{C}S_{C}}\right)\left(\frac{∆H^{\prime }}{K_{H}S_{H}}\right)}$$

The clinically acceptable threshold for color difference (ΔE00) was set at 1.8 based on previously published studies (Paravina et al. 2019). The average ΔE00 values are recorded in the table, and the minimum thickness required to mask the underlying dark color was determined by comparing the measured Δ*E* values and the clinically acceptable ΔE00 (Δ*E* ≤ 1.8).

### 2.5 Statistical Analysis Method

The data analysis for this study utilized SPSS (Statistical Package for the Social Sciences) version 26.0, developed by IBM in Armonk, New York, USA. The research focused on measuring and reporting the average and standard deviation of overall color changes (ΔE00) across various ceramic groups and their respective four thicknesses on the background of C4 and A2 porcelain. To determine the impact of the type of ceramic, its thickness, and its combined effects on ∆E, a two‐way analysis of variance (ANOVA) was employed. Moreover, a one‐way analysis of variance test was conducted to examine the ∆E in bleach shade ceramic groups based on thickness and thickness groups based on ceramic type. Further analysis included two‐by‐two simultaneous comparisons of ceramics in terms of thickness and thickness groups based on ceramic types using Tukey's test. The statistical test was conducted at a 0.05 significance level.

## 3 Findings

### 3.1 Descriptive Findings

Table 2 shows the average and standard deviation of ΔE00 in ceramics for CD, VS, VM, and Zr groups with different thicknesses of 0.5, 0.1, 1.5, and 2.0 mm. According to the findings, only Zr ceramic has ΔE00 < 1.8 at a thicknesses of 0.5 and 1.0 mm. For other thicknesses, all ceramics exhibited ΔE00 ≤ 1.8. The table also indicates that the value of ΔE00 decreases as the thickness of the ceramics increases.

**Table 2:** Average and standard deviation of ΔE00 in ceramics and different thicknesses.

Thickness (mm)					
Ceramic^a^	0.5	1.0	1.5	2.0	*p* value
CD	3.86 ± 0.46 Aa	2.44 ± 0.30 Ab	1.86 ± 0.08 Ac	0.55 ± 0.32 ABd	< 0.001
VS	3.81 ± 0.37 Aa	2.25 ± 0.47 Ab	1.79 ± 0.86 Ab	0.51 ± 0.18 ABc	< 0.001
VM	3.15 ± 0.15 Ba	2.14 ± 0.26 Ab	0.84 ± 0.39 Bc	0.32 ± 0.15 Ad	< 0.001
Zr	1.66 ± 0.20 Ca	0.88 ± 0.39 Cb	0.49 ± 0.12 Bc	0.60 ± 0.19 Bbc	< 0.001
P value	< 0.001	< 0.001	< 0.001	0.043	

*Note:* Indices with capital letters indicate the column‐wise comparison, and small letters indicate the comparison of the values in the row.

^a^ Celtra Duo (CD), Vita Suprinity (VS), Vita Mark (VM), and Zirconia (Zr).

In a one‐way analysis of variance, significant differences were found in the ΔE00 values for thicknesses of 1 mm (*p* < 0.001), 1.5 mm (*p* < 0.001), and 0.5 mm (*p* < 0.001), and 2 mm (*p* = 0.043) across different ceramics. Therefore, significant differences were observed in the ΔE00 values for all thicknesses. This suggests that the type of ceramic affects the ΔE00 value statistically.

**Chart 1:**
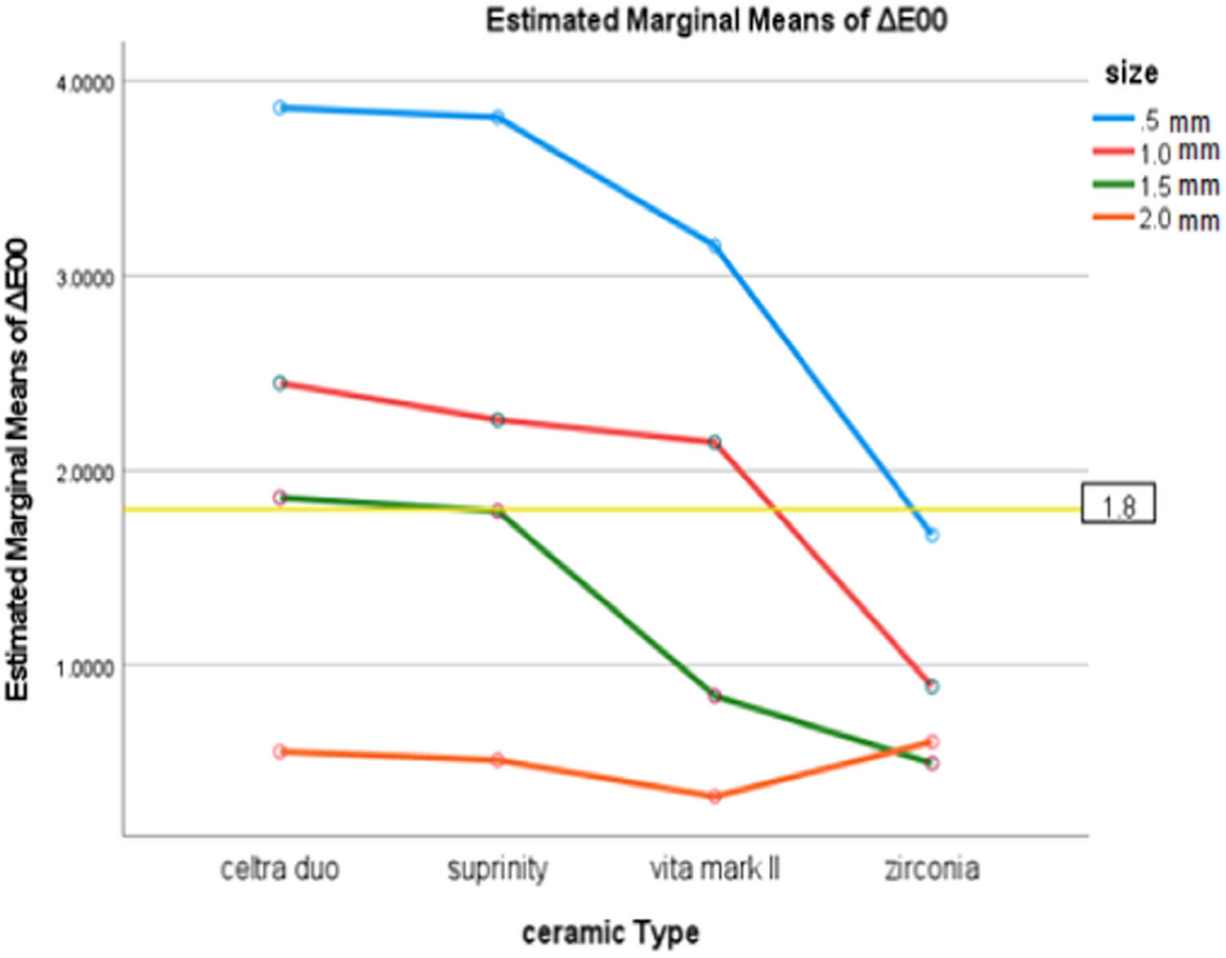
Average changes of ΔE00 based on the thickness and type of ceramic. Note: The Celtra Duo ceramic at a thickness of 1.5 mm showed a ΔE00 value of 1.86, which is marginally above the threshold of 1.8. This indicates that its masking ability can be considered acceptable within a marginal range.

According to the results of a one‐way analysis of variance, significant differences were found in terms of ΔE00 for different thicknesses of a specific type of ceramic (*p* < 0.001). Across all ceramics excluding Zr, the highest ΔE00 (indicating the lowest masking ability) was observed at a thickness of 0.5 mm. In comparison, the lowest ΔE00 (indicating the highest masking ability) was observed at a thickness of 2.0 mm. In the zirconia group, similar to other ceramics, the highest ΔE00 values (indicating the lowest masking ability) was observed at a thickness of 0.5 mm. However, unlike other ceramics, the lowest ΔE00 value (indicating the highest masking ability) was not observed at a thickness of 2.0 mm but rather at 1.5 mm, indicating a distinct behavior in its masking ability. Chart 1 shows the ΔE00 values of ceramics in different thicknesses.

In Chart 1, it is evident that for thicknesses of 1.5 mm and 2.0 mm, almost all types of ceramics exhibit ΔE00 values that fall below the threshold of 1.8.

The ΔE00 value is affected by both the type of ceramic and its thickness. There is a significant interaction effect between the thickness and the type of ceramic on the average value of ΔE00.

According to the results of a two‐way analysis of variance (ANOVA), the effects of ceramic thickness (*p* < 0.001), the type of ceramic (*p* < 0.001), and the interaction effects of ceramic and thickness (*p* < 0.001) on ΔE00 were significant.

## 4 Discussion

Based on the research findings, the null hypothesis was rejected, indicating that the color masking ability of the C4 porcelain substrate varies depending on the thickness and type of ceramics used (Vita Mark II, Celtra Duo, Suprinity, and zirconia). The research revealed that the most significant ΔE00 occurred with a thickness of 0.5 mm, while the most minor ΔE00 were observed with a thickness of 2.0 mm. This suggests that ΔE00 decreases as the thickness of the ceramics increases. Additionally, at a thickness of 0.5 and 1 mm, only zirconia had ΔE00 values below the acceptable threshold, whereas the ΔE00 of the other ceramics exceeded the acceptable threshold.

According to Lambert's law, when a material's thickness is reduced, its absorption decreases, allowing more light to pass through. Conversely, light transmission decreases, and absorption increases as the thickness increases (Nassau 2001). This law explains why the masking ability increases and the Δ*E* values decrease as the thickness of ceramics increases. Other studies have supported this finding (Ongun et al. 2021; Al‐Haj Husain et al. 2020; Chongkavinit and Anunmana 2021).

Fachinetto et al. (2023) investigated the masking ability of monolithic CAD/CAM ceramics in three thicknesses of 0.5, 1.0, and 1.5 mm. The study results showed that the most adequate thickness to mask the discoloration depends on the degree of discoloration. However, less color difference (Δ*E*) values were measured when LT (low‐translucent) ceramics were used with a thickness above 1 mm and white opaque try‐in paste (Fachinetto et al. 2023).

In a study by Ellakany et al. (2021), the masking ability of two types of CAD/CAM ceramics was investigated. The research found that the color difference was highest in ceramics with a thickness of 0.5 mm and lowest in ceramics with a thickness of 1.5 mm (Ellakany et al. 2021). The study concluded that increasing the ceramic thickness significantly impacted the masking ability of the color of the dental substrate, which is consistent with present research.

In the present research, only zirconia could mask discoloration in the thickness of 0.5 mm and 1 mm, but in other thicknesses (1.5 mm and 2 mm), almost all ceramics had ΔE00 ≤ 1.8, which is clinically acceptable.

Alfouzan et al. (2022) examined various ceramics with 1.0 and 2.0 mm thicknesses. Their study demonstrated that all the ceramics analyzed exhibited satisfactory color masking, consistent with the current research findings (Alfouzan et al. 2022).

Vichi et al. (2000) examined the ability of ceramic restorations to match the color of natural teeth at various thicknesses (1.0 mm, 1.5 mm, and 2.0 mm) using Lucite‐reinforced ceramics and different opaque posts. They found complete color matching only at a 2.0‐mm thickness (Vichi et al. 2000). The variation in results is attributed to the differences in ceramic types and backgrounds. Understanding dental restorations relies on the interaction of light with ceramics and their inherent characteristics, including opacity and translucency (Vichi et al. 2000).

The Vita Mark feldspathic ceramic has been reported to have the highest amount of light transmission, while glass‐ceramic reinforced with zirconia (Vita Suprinity) has the lowest (Straumann n.d.). It has been found that ceramics with an initial thickness of 1.0 mm absorb 58.2%–58.7% of the light, and this percentage increases to 64.4%–94.0% for ceramic disks with a thickness of 2.0 mm (Straumann n.d.). As the thickness of the ceramic increases, the amount of absorbed light also increases, improving its color masking ability. However, this also makes the restorations appear more opaque.

The optical properties of ceramic restorations, including light transmission, depending on crystal structure, grain size, pigments, particle volume and distribution, and porosity (Stawarczyk et al. 2016; Ilie and Hickel 2008; Sheibani et al. 2024). Different compositions lead to variations in the masking ability of different colors. For example, lithium disilicate glass‐ceramic samples have lower light transmission due to their different microstructure and denser crystals than feldspathic ceramics (Stawarczyk et al. 2016). Vita Mark ceramic has a less dense microstructure and is made of feldspathic ceramic reinforced with sanidine (KASi_3_O_8_) with a crystalline content of 30% (Sakaguchi and Powers 2012). Ceramics with higher crystalline content, like Vita Suprinity, have different translucency. Zirconium oxide crystals in glass ceramics can reduce light transmission, making the ceramic look more opaque due to zirconium compounds absorbing more light (van Noort 2013). If the crystals in the ceramic composition are smaller than the wavelength of visible light, the glass will appear more transparent. However, reducing light emission and reflection can make the substance more opaque (van Noort 2013). Celtra Duo is a lithium silicate ceramic reinforced with zirconia and contains 10% lithium silicate and zirconium dioxide in its composition. The dissolution of zirconia in the lithium silicate glass matrix results in smaller silicate crystals and higher translucency, which affects masking ability results (Vichi et al. 2000; Antonson and Anusavice 2001; Bagis and Turgut 2013).

Based on specific reports, the ceramic thickness used to conceal tooth discoloration in the final restorations should be at least 2.0 mm (Vichi et al. 2000). Shadman et al. (2015) found that the minimum thickness of multilayer IPS e. max ceramic needed to mask color changes was 0.8 mm, which aligns with the current research results (Shadman et al. 2015). Based on the current research findings, it was observed that ceramics of two different thicknesses (1.5 and 2.0 mm) effectively concealed discolorations, with ΔE00 almost staying below the clinically acceptable threshold of 1.8 in all cases. Notably, color changes were less pronounced in thicker ceramics, particularly in the 2.0‐mm thickness. These results carry significant clinical implications as the increased tooth grinding required to prepare these thicker restorations poses a risk to the pulp's health. The minimum thickness of the ceramics studied in this research for masking blemishes was 1.5 mm. Only the zirconia samples showed acceptable masking ability when the thickness was 0.5 and 1 mm. A previous study reported that the minimum thickness of zirconia to achieve an acceptable color match (Δ*E* < 2.6) was 0.9 mm (Ellakany et al. 2021), which aligns with the results of this study. Tabatabaian et al. (2018) found that the best outcome for masking discoloration (Δ*E* < 2.6) was achieved with a 1.6‐mm thickness (Tabatabaian et al. 2018). Our study observed that zirconia exhibited better masking ability than other ceramics at lower thicknesses, specifically at 0.5 and 1 mm. However, at a higher thickness (i.e., 2.0 mm), the masking ability of zirconia was comparable to that of other ceramics. The choice of 5Y‐TZP zirconia in this study was based on its higher translucency compared to 3Y‐TZP. The increased yttria content (5 mol%) in 5Y‐TZP reduces tetragonal phase grains and increases cubic phase grains, allowing better light transmission and enhancing its esthetic properties. This characteristic makes 5Y‐TZP particularly suitable for monolithic restorations, which do not require veneering with additional translucent ceramic layers. In contrast, 3Y‐TZP, while mechanically superior, is typically recommended for structural purposes, such as copings, due to its lower translucency.

The human eye can detect a color difference (Δ*E*) of 3.7 in dental structures, which is considered a clinically acceptable limit in uncontrolled clinical conditions (Johnston and Kao 1989). However, various factors can affect the ability to detect color differences under different conditions. In standard conditions, the acceptable threshold for color differences in teeth reconstruction as determined by the human eye is 1.8 (Thoma et al. 2016). Based on current research, the clinical acceptance criteria for color differences Is set at 1.8 (Paravina et al. 2019). The findings indicate that ceramic thicknesses of 1.5 and 2.0 mm can effectively conceal discoloration.

This research utilized a dental porcelain substrate of C4 color to mimic severely discolored teeth. The same substrate was also employed in the study by Shadman et al. (2015).

This research aimed to analyze the masking ability of four distinct types of ceramics at varying thicknesses on a dental porcelain substrate with C4 color. It is essential to consider the potential influence of the substrate's color on the final color of the restoration. Therefore, it is crucial to conduct an assessment using different substrate colors. Furthermore, there is a need for more investigation to determine the minimum thickness required for discolored dental masking across diverse ceramic types and colors. Additionally, it would be valuable to explore the degree of translucency exhibited by different ceramics.

## 5 Conclusion

Examining the masking ability of CAD/CAM bleach shade ceramics in different thicknesses over background C4 showed:

The effects of thickness, ceramic type, and the interaction effects of variables on ΔE00 were significant (all three: *p* < 0.001).

By increasing the thickness of the ceramic, their masking ability was improved.

Almost all the investigated ceramics at 1.5 and 2 mm thicknesses could mask discolorations, and ΔE00 was lower than the clinically acceptable threshold (1.8) in all cases. At 0.5 and 1 mm thicknesses, only zirconia had ΔE00 values below the threshold limit, and ΔE00 of other ceramics was higher than the acceptable threshold limit.

Overall, zirconia had better masking ability in all thicknesses.

## Author Contributions

**Shervin Reybod:** data collection, writing the initial draft of the manuscript, editing the manuscript, manuscript revision. **Fariba Ezoji:** reviewing and editing the manuscript. **Ghazaleh Ahmadizenouz:** data analysis and interpretation, reviewing and editing the manuscript. **Behnaz Esmaeili:** study design, supervision, manuscript revision, and final approval of the manuscript.

## Ethics Statement

The research was conducted under laboratory and experimental conditions with no patient interaction. Therefore, there were no specific ethical concerns or issues. Additionally, before the commencement of the research, the execution protocol was reviewed and approved by the Ethics Committee in Medical Research at Babol University of Medical Sciences (Ethics ID: IR. MUBABOL.HRI.REC.1401.276).

## Consent

This study does not involve human participants, so informed consent was not applicable.

## Conflicts of Interest

The authors declare no conflicts of interest.

## Data Availability

The data that support the findings of this study are available from the corresponding author upon reasonable request.
